# Early Expression of Functional Markers on CD4^+^ T Cells Predicts Outcomes in ICU Patients With Sepsis

**DOI:** 10.3389/fimmu.2022.938538

**Published:** 2022-07-11

**Authors:** Jianwei Chen, Hao Wang, Ran Guo, Haolong Li, Na Cui

**Affiliations:** ^1^ Department of Critical Care Medicine, State Key Laboratory of Complex Severe and Rare Diseases, Peking Union Medical College Hospital, Chinese Academy of Medical Science and Peking Union Medical College, Beijing, China; ^2^ Department of Critical Care Medicine, Beijing Jishuitan Hospital, Beijing, China

**Keywords:** sepsis induced immunosuppression, mTOR, IFN-γ, PD-1, T-cell function

## Abstract

**Objective:**

There is evidence that metabolic disorder, dysfunction and abnormal apoptosis of immune cells are closely related to immunosuppression in sepsis. Single monitoring of exhaustion receptors does not reflect well the immune status of septic patients; therefore, we monitored immune status in relation to metabolism, function and apoptosis of immune cells to find good prognostic indicators for sepsis.

**Design:**

A single-center prospective observational study.

**Setting:**

Teaching hospital including an academic tertiary care center.

**Patients:**

81 patients with sepsis and 22 without sepsis admitted to the ICU.

**Interventions:**

Patients were divided according to Sequential Organ Failure Assessment (SOFA) score: mild sepsis 2–5 points and severe sepsis ≥6 points. SOFA score was recalculated daily. If it changed by ≥2 points within 2 days, T-cell metabolism, function and apoptotic makers [mammalian target of rapamycin (mTOR), T-bet, interferon (IFN)-γ, granzyme B, and programmed cell death (PD)-1] were continuously monitored on days 1, 3 and 5 after admission.

**Measurements and Main Results:**

The overall status of immune cells was compared among patients with different severity of sepsis. Patients with severe sepsis, compared with mild and no sepsis, had lower lymphocyte counts, higher expression of receptors associated with cell metabolism, activation and apoptosis, and lower expression of functional receptors. Multivariate regression analysis revealed that frequency of CD4^+^ T cells expressing mTOR, IFN-γ and PD-1 at admission was an independent predictor of 28-day mortality. Receiver operating characteristic curve analysis indicated that frequency of CD4^+^ T cells expressing mTOR, IFN-γ and PD-1 predicted 28-day mortality, with cutoffs of 30.57%, 12.81% and 22.46%, respectively. The expression of related receptors on CD8+ T cells showed similar trend to that on CD4+ T cells, but no significant difference was found.

**Conclusions:**

Abnormally increased expression of metabolic and apoptotic receptors on CD4^+^ T cells and decreased expression of functional factors are associated with poor prognosis in ICU patients with sepsis. Poor prognosis can be identified by early detection of expression of mammalian target of rapamycin (mTOR), IFN-γ and PD-1 on CD4^+^ T cells.

## Introduction

Sepsis, defined as organ dysfunction caused by a dysregulated host response, is responsible for 11 million deaths every year, and accounts for 19.7% of deaths worldwide (1). As the COVID-19 crisis persists, sepsis-related mortality may continue to increase (2). Most patients with sepsis exhibit an excessive inflammatory response – immunosuppressive status and most sepsis mortality is cause by late immunosuppression. Although the causes of immunosuppression in patients with sepsis remain unclear, increasing evidence shows that the underlying reasons may include metabolic disorders, reduced functions, and decreased number in immune cells (3).

T-cell immune dysfunction in patients with sepsis is one of the important factors that lead to serious impairment of immune function. Early recognition of the immune status of T cells in patients with sepsis and an improvement in supportive therapy may contribute to reducing the mortality rate. However, the immune cell count or apoptosis level is unable to accurately reflect the immune status of patients with sepsis. To prospectively determine the systemic immune status of T cells in patients with sepsis, we determined the metabolic activity, killing function, and apoptosis-related receptor expression in CD4^+^ and CD8^+^ T cells.

Mammalian target of rapamycin (mTOR) is a serine/threonine kinase that plays a significant role in T-cell development, homeostasis, activation, and effector cell fate decisions. T cells maintain cell activity *via* mTOR-related signaling to integrate immune and metabolic signals in many conditions (4). T-bet is an important transcription factor that activates effector T cells, and is involved in CD4 and CD8 effector T-cell differentiation and functional cytokine production (5), and its expression is critically regulated by the mTOR signaling pathway (6, 7). mTOR is involved in the regulation of T-cell differentiation *via* regulating expression of T-bet (8), which implies that expression of mTOR and T-bet is an important indicator of T-cell metabolism and activation. Specifically, T-bet promotes secretion of IFN-γ and granzyme B by T cells, which are important killing factors for CD4^+^ and CD8^+^ T cells to exert anti-infective effects (9–11). T cells express negative costimulatory molecules that suppress their function and prevent excessive activation. Programmed cell death (PD)-1 is a receptor expressed by lymphocytes that functions in an inhibitory manner. Blocking the PD-1 pathway may result in impaired T-cell functions, including cytokine production and cytotoxic activity. In sepsis, PD-1 signaling plays an important role in lymphocyte exhaustion, and blockade of the PD-1 pathway improved survival in a mouse model of sepsis (12). The five receptors mentioned above play important roles in the execution of different functions by T cells. Therefore, the percentage of T cells expressing related receptors indirectly reflects the status of T cells, including metabolism, activation, apoptosis and immune function.

**Figure 1:**
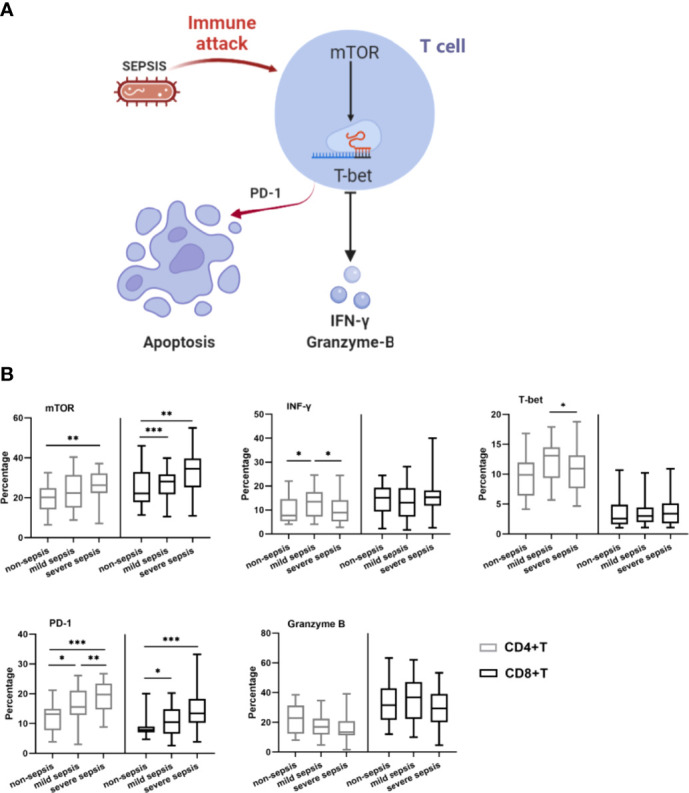
Characterization of immune cell subsets. **(A)** Schematic of mTOR-mediated T cell apoptosis caused by immune attack in sepsis. **(B)** The lymphocyte count and immune cell subset of the study population. Expression of IFN-γ, PD-1, T-bet, mTOR and Granzyme B on CD4^+^T cells and CD8^+^ T cells in control group, mild and severe group were measured on day 1 at the onset of sepsis. Data are shown as box plot with max and minimum. *p < 0.05; **p < 0.01; ***p < 0.001.

Based on previous animal studies, we hypothesized that a similar pathway may exist in human lymphocytes (**Figure 1A**). Eighty-one patients with differing severity of sepsis and 22 patients without sepsis in ICU were continuously monitored for immune parameters, including absolute number of CD4^+^ and CD8^+^ T cells, and percentage of CD4^+^ and CD8^+^ T cells expressing mTOR, IFN-γ, PD-1 and granzyme B. This study aimed to investigate the possible mechanisms of the development of immunosuppression in patients with sepsis and the relationship of these mechanisms with prognosis by detecting changes in the number and function of immune cells.

## Materials and Methods

### Patients and Study Design

This was a prospective study conducted between April 2020 to December 2021 in Peking Union Medical College Hospital (PUMCH), China. The study was conducted in accordance with the amended Declaration of Helsinki and approved by the ethics committee of PUMCH(JS-2800). Written informed consent was obtained from all patients.

We prospectively included 81 adult septic patients and 22 non-septic patients in ICU. Inclusion criteria were as follows: (1) age≥18 years, (2) ICU stay time≥48 h, and (3) diagnosis of sepsis. Exclusion criteria were (1) age < 18 years, (2) pregnant or lactating, and (3) had immunosuppressive host factors (autoimmune disease or receiving immunosuppressive medication). The control group of patients was composed of critically ill, non-septic patients (controls).

According to the SOFA score, septic patients were divided into Mild sepsis Group (SOFA 2-5) and Severe sepsis Group (SOFA ≥ 6). Septic patients’ SOFA scores were calculated daily, and the expression of T cell function markers were continuously monitored if patients’ SOFA scores were found to change by ≥ 2 points within 2 days.

### Clinical and Laboratory Evaluation

At admission to the ICU, all patients underwent comprehensive clinical assessment: age, sex, and underlying diseases. Acute Physiology and Chronic Health Evaluation (APACHE) II and Sequential Organ Failure Assessment (SOFA) scores were calculated. Organ functional and inflammatory parameters were determined immediately by PUMCH laboratories. Besides, life-sustaining treatments, biochemical parameters, treatment-related factors were recorded daily. During follow-up the following data were collected: the duration of ICU stay and in-hospital stay, ICU and in-hospital mortality, and 28-day mortality.

### Flow Cytometry

Peripheral blood samples were collected in EDTA anticoagulant tubes on the first day following ICU admission, peripheral blood mononuclear cells (PBMCs) were isolated using FICOLL density gradient separation method. After that, PBMCs (1x10^6^/ml) were fixed and permeabilized using a Fix/Perm Buffer Set (426803, Biolegend), then stained with combinations of fluorochrome-conjugated antibodies.

Antibodies were purchased from Bio Legend (San Diego, CA) and Invitrogen (California, United States). According to the manufacturer’s instructions, PBMCs were stained and their isotype controls at 4°C for 30 min, then washed with staining buffer 3 times (See attached list of antibodies). Flow cytometry analysis was performed with flow cytometer (muti-color Cytek Aurora flow cytometer, Cytek Biosciences, Fremont, CA) and analyzed using the FlowJo software (v.10.1r5, TreeStar). Lymphocytes were gated by side scatter (SSC) and forward scatter (FSC), T-cells (CD3^+^), CD4^+^ and CD8^+^ T-cell subsets were quantitated, and mTOR, T-bet, Granzyme B and IFN-γ were further quantitated on CD4^+^ and CD8^+^ T cells. At least 100000 cells were analyzed from each sample.

### Statistical Analysis

After testing for normal distribution with the Shapiro–Wilk normality test, normally distributed data were expressed as mean ± standard deviations (SD), statistical significance was determined using Student t-test or one-way ANOVA. Nonnormally distributed data were expressed as the median and interquartile range, statistical significance was determined using Mann-Whitney U test or Kruskal Wallis test. Categorical variables were presented as proportions and analyzed using the w2 or Fisher exact test. Multivariate logistic regression analyses were performed to determine independent risk factors for 28-day mortality, the results were expressed as P and odds ratio (OR) with 95% confidence interval (CI). Receiver–operating characteristic (ROC) curves were used to distinguish diagnostic value of immune parameters to 28-day mortality. Kaplan–Meier survival data were performed to assess time to 28-day mortality and analyzed using two-sided log-rank test. Statistical analyses were performed using the SPSS 21.0(Chicago, Illinois) and GraphPad Prism 8.0 (San Diego, CA). Significant p-values are represented as *p < 0.05; **p < 0.01; ***p < 0.001.

## Results

### Patients and Baseline Characteristics

Within the study period, 128 patients admitted to ICU were screened initially. 20 of them satisfied at least one of the exclusion criteria, 3 of them refused to sign the consent form, and 2 were lost to follow-up. Finally, a total of 103 patients were enrolled in the study, including 22 non-septic patients and 81 septic patients (**Supplement Figure 1**). **Table 1** shows the main characteristics of patients at ICU admission. No differences were identified among the groups in age, sex, comorbidities at ICU admission (p > 0.05). Septic patients have marked higher APACHE II score (*P*=0.001) than non-septic patients, and severe septic patients have higher APACHE II score than mild septic patients. Compared to the non-sepsis patients, the proportion of patients with bacteria infection (P=0.00) and vasopressor usage (p =0.004) was significantly higher in the septic population, besides, the level of creatinine (P=0.006) and TBiL (P=0.001) was also higher. As for the prognosis related parameters, severe septic patients had longer ICU times (P<0.001) and higher ICU mortality (p=0.006), 28-day mortality (P=0.023) than mild septic and non-septic patients.

**Table 1:** Characteristics of patients at ICU admission.

Variables	Non-sepsis (control subjects, n=22)	Septic patients		P-value
		Mild sepsis (SOFA 2-5, n=42)	Severe sepsis (SOFA≥6, n=39)	
**Baseline characteristics**				
Age (years)	54 (26)	65.5 (21.8)	69 (19)	0.052
Sex (male: female)	17:5	24: 18	26: 13	0.266
APACHE II score	12 (3.3)	17 (9.3)	18 (8)	0.001
**Comorbidities (n, %)**				
Heart failure	3 (13.6)	9 (21.4)	10 (25.6)	0.582
COPD	3 (13.6)	3 (7.1)	1 (2.6)	0.245
Diabetic mellitus	5 (22.7)	14 (33.3)	11 (28.2)	0.666
Liver Cirrhosis	2 (9.1)	0 (0)	3 (7.7)	0.096
Tumor	2 (9.1)	10 (23.8)	11 (28.2)	0.226
Chronic renal failure	2 (9.1)	9 (21.4)	10 (25.6)	0.327
**Sites of infection (n, %)**				
Lungs	0 (0)	30 (71.4)	30 (76.9)	0.457*
BSI	1 (4.5)	7 (16.7)	6 (15.4)	0.459
Intra-abdominal	0 (0)	7 (16.7)	13 (33.3)	0.069*
Soft tissue	2 (9.1)	2 (4.8)	4 (10.3)	0.635
Others	0 (0)	4 (9.5)	6 (15.4)	0.322*
**Pathogens (n, %)**				
Bacteria	5 (22.7)	34 (81)	33 (84.6)	0.000
Fungal	0 (0)	11 (26.2)	13 (33.3)	0.627*
Virus	0 (0)	1 (2.4)	4 (10.3)	0.157*
Else	2 (9.1)	2 (4.8)	2 (5.1)	0.748
**Laboratory test at admission**				
Creatinine (umol/L)	76.5 (27)	95 (95.5)	136 (136)	0.006
Albumin (g/L)	32.5 (5.5)	31 (4)	31 (5)	0.099
TBiL (umol/L)	12.7 (15.6)	16.8 (20.2)	29.9 (39.6)	0.001
**Life-sustaining treatments (n, %)**				
Mechanical ventilation	21 (95.5)	36 (85.7)	36 (92.3)	0.515
Need for vasopressor	7 (31.8)	18 (42.9)	28 (71.8)	0.004
Need for RRT	5 (22.7)	8 (19)	16 (41)	0.073
**Prognosis parameters**				
ICU durations	4 (3.5)	11 (10.3)	11 (12)	< 0.001
ICU mortality **(n, %)**	0 (0)	3 (7.1)	10 (25.6)	0.006
Hospital durations **(day)**	18 (7)	13 (20)	16 (22)	0.379
Hospital mortality **(n, %)**	2 (9.1)	5 (11.9)	11 (28.3)	0.1
28 Day mortality **(n, %)**	0 (0)	4 (9.5)	9 (23.1)	0.023
**Inflammatory markers**				
PCT (ng/mL)	1.95 (4)	0.64 (2)	2 (10)	0.019
CRP (mg/dL)	96.1 (60.59)	106.45 (103.61)	118.94 (158.30)	0.376
IL-6 (pg/ml)	69 (188)	31.4 (65)	37 (89)	0.095
IL-8 (pg/ml)	73 (125)	80 (90)	134 (158)	0.039
IL-10 (pg/ml)	9,5 (17.6)	5.9 (10.1)	8.4 (24)	0.334
TNF-α (pg/ml)	11 (4.95)	12.6 (10)	19 (12)	0.001
**Immune parameters**				
C3 (g/L)	0.78 (0.27)	0.83 (0.28)	0.92 (0.73)	0.091
C4 (g/L)	0.16 (0.08)	0.17 (0.11)	0.18 (0.17)	0.563
IgA (g/L)	2.23 (1.38)	2.05 (1.61)	2.35 (2.24)	0.618
IgG (g/L)	8.25 (3.93)	8.2 (4.56)	7.52 (4.38)	0.118
IgM (g/L)	0.55 (0.84)	0.61 (0.60)	0.58 (0.38)	0.912
**Immune cell subsets (cells/μl)**				
WBC (cells/mm^3^)	12095 (4810)	8770 (6500)	10020 (7580)	0.058
NG (cells/mm^3^)	10.32 (3.86)	7.08 (6.91)	8 (7.58)	0.13
NK (cells/mm^3^)	49.5 (113)	63 (58)	81 (101)	0.862
Lymphocyte (cells/mm^3^)	1014 (520)	971 (667)	781 (622)	0.032
T cells	697.7 ± 342.4	681.63 ± 378	556.4 ± 258.1	0.025
B cells	147.5 (173.5)	101.5 (170.3)	63 (148)	0.076

APACHE II, Acute Physiology and Chronic Health Evaluation II; COPD, chronic obstructive pulmonary disease; BSI, blood steam infection; TBIL, total bilirubin; RRT, renal replacement therapy; SOFA, Sequential Organ Failure Assessment. PCT, procalcitonin; CRP, C-reactive protein; IL-6, interleukin-6; IL-8, interleukin-8; IL-10, interleukin-10; TNF-α, Tumor necrosis factor; C3, complement factor 3; C4, complement factor 4; IG, immunoglobulin; WBC, white blood cell; NG, neutrophilic granulocyte; NK, natural killer cell; LY, Lymphocyte. Continuous variables are expressed as the median and interquartile range, other data are raw numbers (%). P value for the comparison between Non-sepsis, Mild Sepsis and severe Sepsis. P* refers to P values between Mild Sepsis and severe Sepsis.

### Comparison of Immune Parameters Among 3 Groups

PCT (p=0.019), IL-8 (p=0.039) and TNF-α (p=0.001) were significantly higher in severe sepsis group than mild and non-septic group. There were no significant differences in other immune parameters among the 3 groups. As for immune cell subsets, lymphocytes (p=0.032) and T lymphocytes (p=0.025) absolute numbers were significantly higher in control group (**Table 1**).

We compared the expression of T cell function makers among the three groups at admission (**Figure 1B** and **Supplement Table 1**). CD4 ^+^T (P=0.002) and CD8^+^T cell (P=0.004) were significantly lower in septic patients. The percentage of CD4^+^T cells expressing mTOR, T-bet, IFN-γ and PD-1 were significantly higher in septic population than that in non-septic patients (p=0.012, 0.005, 0.044, <0.001, respectively). A corresponding increase in the expression of mTOR (P=0.002) and PD-1(P<0.001) on CD8^+^T cells were also found in septic patients. Interestingly, the mild sepsis group CD4^+^T cell had higher expression of T-bet and IFN-γ than severe populations. While the Granzyme B expression ratio on CD4^+^T cells was lower in severe sepsis patients (P=0.026).

### Comparison of Immune Parameters According to 28-Day Mortality

We divided septic patients into survivors and non-survivors according to 28-day prognosis. Immune related parameters between the two groups are shown in **Table 2**. Higher level of TNF-a (p=0.03) and lower absolute number of T cells, CD4^+^T and CD8^+^T (p=0.037, 0.046, 0.043, respectively) were observed in non-survivors. As for functional phenotype of T cells, compared with survivors, the percentages of CD4^+^T cell expressing mTOR (P=0.026) and PD-1(P=0.015) were significantly higher in non- survivors, while the expression of T-bet (P=0.019) and IFN-γ (P=0.001) were lower. Besides, CD8^+^T cell expressing PD-1 (P=0.012) were also higher in non-survivors.

**Table 2:** Comparison in Immune biomarkers of septic patients based upon 28-day mortality.

Variables	Overall population (n=81)	Survivors (n=68)	Non-survivors (n=13)	P value
**Inflammatory markers**				
PCT (ng/mL)	1.3 (5)	0.97 (4)	3.9 (11)	0.06
CRP (mg/dL)	119.29 (132.55)	106.451 (08.61)	166.62 (165.19)	0.077
IL-6 (pg/ml)	35.8 (80.13)	34.55 (88.93)	50.15 (74.65)	0.532
IL-8 (pg/ml)	101 (109.5)	97.5 (112.5)	123 (206)	0.483
IL-10 (pg/ml)	5.95 (12.98)	5.9 (14.4)	9.05 (8.8)	0.413
TNF-α (pg/ml)	16.3 (13.2)	16 (13)	23.6 (14)	0.03
**Immune parameters**				
C3 (g/L)	0.871 (0.385)	0.87 (0.401)	0.907 (0.463)	0.949
C4 (g/L)	0.18 (0.12)	0.18 (0.156)	0.153 (0.105)	0.088
IgA (g/L)	2.19 (1.47)	2.29 (2)	2.58 (2)	0.748
IgG (g/L)	8.05, 4.3	7.63 (4.72)	8.84 (7.75)	0.463
IgM (g/L)	0.61 (0.48)	0.62 (0.6)	0.5 (0.45)	0.081
**Lymphocyte subsets**				
WBC (cells/mm3)	9240 (7462.5)	9355 (7667)	7400 (6543)	0.169
NG (cells/mm3)	7.53 (8.11)	7.1 (7.89)	7.59 (8.85)	0.364
NK (cells/mm3)	62 (75.3)	78.5 (84)	41 (61)	0.066
LY (cells/mm3)	742 (639.5)	878 (650.5)	633 (417.8)	0.068
CD3^+^T (cells/mm3)	572.56 ± 247.1	627.6 ± 290.7	448.6 ± 195	0.037
CD4^+^T (cells/mm3)	303.1 ± 80.15	310 ± 78.94	260.84 ± 87.08	0.046
Percentage of mTOR^+^/CD4^+^ T cells (%)	23.97 ± 8.87	23.69 ± 9.83	29.6 ± 6.5	0.026
Percentage of T-bet^+^/CD4^+^ T cells (%)	12.41 (5.56)	12.46 (5.24)	9.5(5.71)	0.019
Percentage of IFN-γ^+^/CD4^+^ T cells (%)	12.06 (9.83)	13.27 (10.11)	5.24 (7.08)	0.001
Percentage of Granzyme B^+^/CD4^+^ T cells (%)	16.59 ± 7.64	17.04 ± 8.33	15.47 ± 5.31	0.516
Percentage of PD-1^+^/CD4^+^ T cells (%)	17.67 ± 5.38	17.04 ± 5.30	20.97 ± 4.76	0.015
CD8^+^T	172 (149.3)	180 (159.5)	150 (100)	0.043
Percentage of mTOR^+^/CD8^+^ T cells (%)	29.79 ± 8.64	29.52 ± 9.17	30.92 ± 7.09	0.604
Percentage of T-bet^+^/CD8^+^ T cells (%)	3.01 (2.99)	3.12 (3.18)	2.84 (1.558)	0.748
Percentage of IFN-γ^+^/CD8^+^ T cells (%)	14.3 (9.7)	15.3 (9.95)	14.81 (11.44)	0.969
Percentage of Granzyme B^+^/CD8^+^ T cells (%)	32.73 ± 14.75	32.65 ± 13.77	30.99 ± 11.44	0.648
Percentage of PD-1^+^/CD8^+^ T cells (%)	12.5(8.36)	11.25 (7.83)	15 (8.35)	0.012
CD4^+^T/CD8^+^T	1.65 (1.36)	1.59 (1.38)	2.18 (1.9)	0.361
B cells (cells/mm3)	84 (171.25)	90.5 (150)	55 (85)	0.285

PCT, procalcitonin; CRP, C-reactive protein; IL-6, interleukin-6; IL-8, interleukin-8; IL-10, interleukin-10; TNF-α, Tumor necrosis factor; C3, complement factor 3; C4, complement factor 4; Ig, immunoglobulin; WBC, white blood cell; NG, neutrophilic granulocyte; NK, natural killer cell; LY, Lymphocyte. Continuous variables are expressed as the median and interquartile range, other data are raw numbers (%). P value for the comparison between Survivor and Non- Survivor group.

Univariate and multivariable logistic regression analysis was conducted for indicators that demonstrated P <0.05 between survivors and non-survivors. Multivariable logistic regression analysis identified mTOR^+^/CD4^+^T% (OR 1.211, 95% CI, 0.71- 0.96, P=0.012), IFN-γ^+^/CD4^+^T% (OR 0.787, 95% CI, 1.049- 1.541, p=0.014) and PD1^+^/CD4^+^T% (OR 1.368, 95% CI, 0.571- 0.937, P=0.013) as independent risk factors for 28-day mortality (**Table 3**).

**Table 3:** Multivariable logistic regression analysis on 28-day mortality.

Parameter	OR	95% CI	P value
mTOR^+^/CD4^+^T%	1.211	1.042-1.408	0.012
IFN-γ^+^/CD4^+^T%	0.787	0.649-0.953	0.014
PD1^+^/CD4^+^%	1.368	1.067-1.752	0.013
T-bet^+^/CD4^+^%	0.753	0.540-1.051	0.095
PD1^+^/CD8^+^%	1.051	0.893-1.237	0.548
T cell	0.999	0.994-1.005	0.824
CD4^+^T	0.990	0.973-1.007	0.262
CD8^+^T	0.995	0.981-1.008	0.435

CI, confidence interval; IFD, OR, odds ratio.

**Figure 2:**
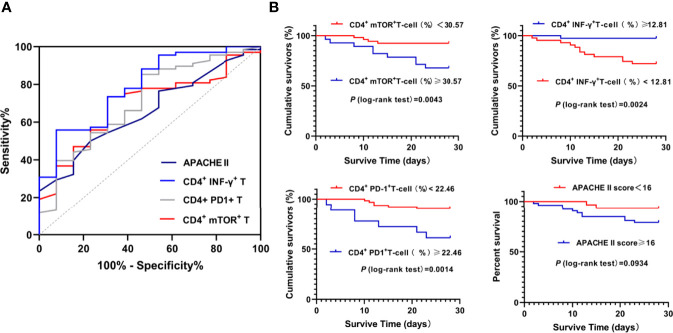
ROC analysis and Kaplan-Meier analysis of parameters predicting D28 Mortality. ROC analysis of parameters predicting D28 Mortality **(A)** and Kaplan-Meier analysis of survival probabilities in Septic study population **(B)** were shown.

To evaluate the distinguishing ability of 28-day mortality, ROC analysis was performed. APACHE II was used as a control indicator. ROC curve analysis showed that mTOR^+^/CD4^+^T% (Area under the curve [AUC], 0.695, 95% CI, 0.582-0.792, P =0.008), PD1^+^/CD4^+^T% (0.71, 0.599-0.806, P=0.01), IFN-γ^+^/CD4^+^T% (0.790,0.685-0.872, P <0.001) had a better discriminatory ability than APACHE II (0.66,0.546-0.761, P =0.033). The cutoff values of mTOR^+^/CD4^+^T%, PD1^+^/CD4^+^T%, IFN-γ^+^/CD4^+^T%, CD4^+^Tcell and APACHE II to predict 28- day mortality were 30.57%, 22.46%, 12.81%, 242.0 and 16.0, with sensitivity of 69.23%, 53.85%, 92.31%, 53.85% and 76.92%, and specificity of 73.53%, 85.29%, 55.88%, 80.88% and 50%, respectively (**Figure 2A** and **Supplement Table 3**). Based above, the Kaplan-Meier survival curve (**Figure 2B**) was performed, mTOR^+^/CD4^+^T cell≥30.57% (log-rank test; P =0.0043), IFN-γ^+^/CD4^+^T cell<12.81% (log-rank test; P =0.0024) and PD-1^+^/CD4^+^T cell≥22.46% (log-rank test; P =0.0014) were associated with lower survival probabilities in our study population.

### Trend of Lymphocyte Receptor Expression in Septic Patients

**Figure 3:**
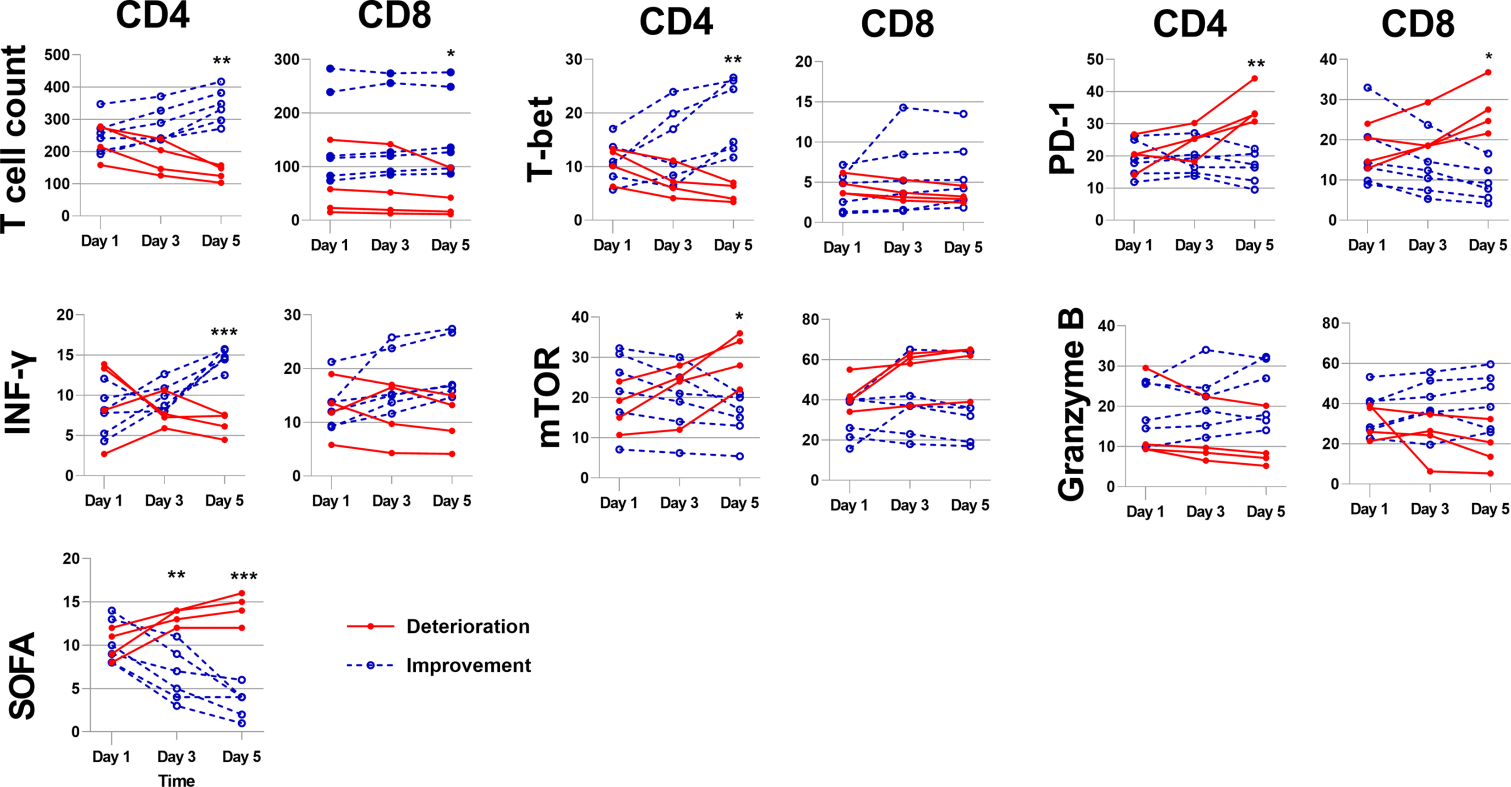
The immune parameters of septic patients were continuously monitored. Changes mTOR, T bet, PD-1, IFN-γ and Granzyme B expression by CD4+ T cells and CD8^+^ T cells between days 1, day 3 and 5 after the admission to the ICU for sepsis. *p < 0.05; **p < 0.01; ***p < 0.001.

The SOFA scores of septic patients were calculated daily, if a patients’ SOFA score changes by ≥ 2 points within 2 days, the function markers expression by T cells will be monitored at day 1, day3 and day 5. A total of 10 patients were monitored continuously, according to changing trends of disease status, the ten patients were divided into improvement group and deterioration group. Compared with day 1, mTOR and PD-1 expression by CD4^+^ T in improvement group was significantly decreased on day 3 and day 5, consistently, the percentage of CD8^+^T expressing mTOR and PD-1 showed the same trend, while T cell count and IFN-γ and T-bet expression by CD4^+^ T gradually increased on day 3 and day 5; these parameters in deterioration group have completely opposite trends compared with improvement group. The Granzyme B expression by CD4^+^T, and the mTOR, IFN-γ, T-bet, Granzyme B expression by CD8^+^ T also changed over time, but no significant difference was found between the two groups (**Figure 3** and **Supplement Figure 2**).

### Correlation Analysis of Key Immune Parameters

As the expression of mTOR, T-bet and IFN-γ, and PD-1 by CD4^+^T cell fluctuates with sepsis severity, and the four markers displayed significant predictive significance for prognosis, which is consistent with our previous reported results. Based above, data of the 10 continuously monitored patients on day 1,3,5 were analyzed by Pearson correlation (**Supplement Figure 3**). The results showed that the percentage of CD4^+^T expressing mTOR in septic patients was negatively correlated with T-bet (r = -0.385; P =0.0359) and IFN-γ (r = -0.363; P =0.049), while it is positively correlated with PD-1 (r = 0.374; P =0.042). In addition, we observed an inverse correlation between the percentage of CD4^+^T expressing T-bet and PD-1 (r = -0.393; P =0.0317), and a strong positive correlation between CD4^+^T expressing T-bet and IFN-γ (r = 0.517; P =0.0036).

## Discussion

Immunosuppression is an important cause of the deficit in controlling the primary infection and susceptibility to secondary infections, and is a common cause of death in septic patients. As an important part of the immune system, impaired cellular immune function further weakens the body’s ability to resist infection, which is reflected in the reduced number and function of T cells. with the proposition of immunosuppression, researchers believe that modulating the host’s immune response to infection contributes to improved outcome in patients with sepsis (3). A recent study reported that combined treatment with IL-7 and anti-PD-1 reversed sepsis-induced T-cell exhaustion and improved survival in patients with sepsis (13, 14). However, because of the lack of standardized multiscale analysis of immune cell phenotype, it is hard to carry out targeted individualized treatment for patients with sepsis. This means that identification of patients with sepsis who could actually benefit from immunotherapy remains unpractical, and this is one of the reasons why immunosuppressive treatments are always promising but seemingly disappointing.

We studied 81 patients with sepsis and 22 without sepsis who were admitted to the ICU. T-cell number and the expression of important receptors implicated in regulating T-cell function, including mTOR, PD-1, T-bet, IFN-γ and granzyme B, were monitored. According to severity, patients were divided into three groups (control, SOFA score <2; mild sepsis, SOFA score 2–5; and severe sepsis, SOFA score ≥6). It should be noted here that the severe sepsis group in this study refers to patients with sepsis with SOFA score≥6, and is not the Severe Sepsis in Sepsis 1.0 (Sepsis patients with organ failure). There were clear differences in immune status among the groups. Patients with or without sepsis, and patients with different severity of sepsis showed distinct immunophenotypes, as demonstrated by lymphocyte count and CD4^+^ T-cell expression of metabolic, functional and apoptotic receptors. ROC curve and multivariate logistic regression analyses found that lymphocyte phenotypic markers were more predictive than cell count for the prognosis of sepsis.

The total number of lymphocytes routinely detected in clinical practice includes memory, central, effector, and other types of lymphocytes. The effector cells play a major role in the fight against infection, so the total number of lymphocytes routinely measured does not accurately reflect immune status. The expression of lymphocyte exhausted maker has a relationship with opportunistic infection and poor prognosis in patients with sepsis, but the ability of a single indicator to predict prognosis is limited (15). In this study, immune monitoring was carried out in relation to metabolic activation, function, and apoptosis of lymphocytes.

In terms of metabolism and cell activation, expression of mTOR on CD4^+^ and CD8^+^ T cells in the sepsis group was significantly higher than that in the non-sepsis group. mTOR is a kinase that is inhibited under normal homeostasis. Its elevated expression in the early stage of sepsis indicates that lymphocytes are stimulated by infection and their metabolism elevated. mTOR expression was higher in the severe sepsis than in the mild sepsis group, suggesting higher levels of lymphocyte mobilization and metabolism in patients with severe sepsis, which seemed to be confirmed by higher levels of IL and TNF-α. However, the metabolic demands of T cells are high and comparable to those of cancer cells (16). Excessive metabolic levels can lead to overload of cell activity, resulting in accumulation of intracellular reactive oxygen species, disruption of intracellular equilibrium, and even early apoptosis (17). Compared with the survival group, early T-cell expression of mTOR was significantly higher in the non-survival group, especially in CD4^+^ T cells, which was consistent with the results of our previous basic research.

Under stressed conditions, T-cell metabolism is highly regulated. However, it is not that T-cell activation leads to upregulation of metabolism, but rather that an increase in the metabolic machinery is an integral component of T-cell activation (18). T-cell activation initiates transcription and translation to generate killer factors needed by the body to resist infection, and T-bet plays a key role in these processes (11). The proportion of lymphocytes expressing T-bet in patients with sepsis was significantly higher than that in patients without sepsis. This suggests that patients with sepsis activate more lymphocytes involved in fighting infection. The proportion of lymphocytes expressing T-bet in patients with SOFA score ≥6 was lower than in patients with SOFA score 2–5. The reasons behind this may be related to the serious immunosuppression in patients with severe sepsis, which was shown by the lower lymphocyte counts in patients with SOFA score ≥6. With the aggravation of sepsis, the expression level of T-BET gradually decreased, which was consistent with Bai et al. (19). Consistent with CD4^+^ T cells, the percentage of T-bet^+^/CD8^+^ T cells in patients with sepsis was also higher than that in non-sepsis patients. However, the percentage of CD8^+^ T cells expressing T-bet was lower than that of CD4^+^ T cells. This is different from patients with CMV, HIV and other virus infections (20, 21). It may be because CD8^+^ T cells, which play an important role in viral infections, are not as important in the response of patients with sepsis, which is mainly caused by bacterial infection. The proportion of patients infected with viruses in our study was small. Correspondingly, expression of IFN-γ and granzyme B in CD8^+^ T cells did not differ significantly among our groups.

In terms of cell function, IFN-γ expression in CD4^+^ T cells in patients with sepsis was higher than that in non-sepsis patients, but IFN-γ expression was lower in the severe sepsis group compared with the mild sepsis group. This is consistent with the trend in expression of T-bet, because it is a strong stimulator of IFN-γ expression (22). Expression of T-bet and IFN-γ in lymphocytes in the non-survival group was significantly lower than that in the survival group, and granzyme B expression was also lower. This means that the secretory function of lymph in the non-survival group was significantly reduced, and accordingly, the ability to resist infection was also reduced. Compared with the mild sepsis and survival groups, although the proportion of T cells expressing granzyme B in the severe sepsis and non-survival groups was lower, there was no significant difference. Granzyme B is mainly secreted by CD8^+^ T cells (15), which may be related to the limited role of CD8^+^ T cells. PD-1, as a marker of cell exhaustion, increased significantly in the severe sepsis and non-survival groups. In the survival and non-survival groups, the changing trend in CD4^+^ and CD8^+^ T cell counts was completely opposite to that of PD-1 expression, suggesting upregulated apoptosis in the non-survival patients.

Multivariate regression analysis indicated that the increased level of mTOR and PD1, as well as reduced level of IFN-γ in CD4^+^ T cells were independent risk factors for 28-day mortality in patients with sepsis. These markers also showed good prognostic predictive ability in the ROC curve analysis. The results implied that abnormally elevated levels of lymphocyte metabolism, decreased anti-infective capacity, and abnormal apoptosis were associated with increased 28-day mortality and deteriorated outcomes. For further validation, we enrolled 10 patients (≥2 points change in SOFA score) from 103 sepsis patients. The CD4^+^ T cell count in the deterioration group was significantly reduced, while expression of mTOR and T-bet, markers reflecting metabolism and activation levels, was increased. IFN-γ, which reflects the killing function, was significantly decreased, and PD-1 was significantly increased, while the improvement group showed the opposite trends. This suggests that the functional status of CD4^+^ T cells is closely related to severity of sepsis. Consistently, a recent study found that expression of inhibitory factors in lymphocytes of patients with sepsis was associated with prognosis (23). This confirms that the functional status of CD4^+^ T cells is related to prognosis.

A limitation of this study was that we only examined a small number of patients, and particularly those that were continuously monitored. This means that the lymphocyte immunophenotypes in patients with sepsis require validation in a larger sample size, and in a multicenter study. Although we found that expression of functional receptors in T cells differed with the severity of sepsis, we failed to clarify the mechanism involved in the changes and the regulatory relationship of them in patients with sepsis. Therefore, further validation in animal models and clinical trials is necessary for further characterization.

## Data Availability Statement

The raw data supporting the conclusions of this article will be made available by the authors, without undue reservation.

## Ethics Statement

The studies involving human participants were reviewed and approved by the Ethics Committee of PUMCH. The patients/participants provided their written informed consent to participate in this study.

## Author Contributions

JC: methodology, visualization, and writing - original draft preparation. RG: data curation and investigation. HL: visualization. HW: project administration and supervision. NC: conceptualization, and writing - review & editing. All authors contributed to the article and approved the submitted version.

## Funding

The work was supported by National Natural Science Foundation of China (No. 82072226), Beijing Municipal Science and Technology Commission (No. Z201100005520049), CAMS Innovation Fund for Medical Sciences (CIFMS) 2021-I2M-1-062 from Chinese Academy of Medical Sciences, National Key R&D Program of China 2021YFC2500800 from Ministry of Science and Technology of the People’s Republic of China.

## Conflict of Interest

The authors declare that the research was conducted in the absence of any commercial or financial relationships that could be construed as a potential conflict of interest.

## Publisher’s Note

All claims expressed in this article are solely those of the authors and do not necessarily represent those of their affiliated organizations, or those of the publisher, the editors and the reviewers. Any product that may be evaluated in this article, or claim that may be made by its manufacturer, is not guaranteed or endorsed by the publisher.
