# Supplementary material for: Early Expression of Functional Markers on CD4+ T Cells Predicts Outcomes in ICU Patients With Sepsis
Source: Front Immunol. 2022 Jul 11;13:938538. doi: 10.3389/fimmu.2022.938538 (PMC9309518; doi:10.3389/fimmu.2022.938538)
Supplement: Supplementary file 1 [file DataSheet_1.zip › Supplement table 1.docx]

**Supplement table 1. Comparison in T cell function makers of the patients at ICU admission**

| **Variables** | **Non-sepsis (control subjects, n=22)** | **Mild sepsis**  **(SOFA 2-5, n=42)** | **Severe sepsis (SOFA≥6, n=39）** | **P value** |
| --- | --- | --- | --- | --- |
| CD4+T (cells/mm3) | 377 (159.3) | 320 (86.8) | 278 (155) | 0.002 |
| Percentage of mTOR+ /CD4+ T cells (%) | 20.16 (26.14) | 22.3 （16.05） | 26.2 （9.84） | 0.012 |
| Percentage of T-bet+ /CD4+ T cells (%) | 9.89(5.55) | 12.97 (5.25) | 11.12 (5.64) | 0.005 |
| Percentage of IFN-γ+ /CD4+ T cells (%) | 7.68 (9.23) | 13.47 (9.77) | 8.84 (9.87) | 0.044 |
| Percentage of Granzyme B+ /CD4+ T cells (%) | 22 ± 19.09 | 18.01 ± 8.18 | 16.2 ± 9.3 | 0.026 |
| Percentage of PD-1+ /CD4+ T cells (%) | 11.83±4.64 | 16.16 ± 5.72 | 18.89±4.85 | < 0.001 |
| CD8+T (cells/mm3) | 211 (255.5) | 202.5 (121.3) | 150 (165) | 0.004 |
| Percentage of mTOR+ /CD8+ T cells (%) | 25.01 ± 9.87 | 27.07 ± 7.63 | 32.53 ± 9.11 | 0.002 |
| Percentage of T-bet+ /CD8+ T cells (%) | 2.58 (3.29) | 3.01 (2.47) | 3.4 (3.32) | 0.746 |
| Percentage of IFN-γ+ /CD8+ T cells (%) | 15.11 (10.06) | 13.6 (12.65) | 15.3 (5.8) | 0.567 |
| Percentage of Granzyme B+ /CD8+ T cells (%) | 33.49 ± 14.67 | 35.16 ± 14 | 29.98 ± 11.69 | 0.186 |
| Percentage of PD-1+ /CD8+ T cells (%) | 7.85 (1.94) | 10.85 (9.44) | 14 (10) | < 0.001 |
| CD4+T/CD8+T | 1.61 (1.32) | 1.53 (1.25) | 2.01 (2.63) | 0.177 |

mTOR, mammalian target of rapamycin; T-bet, T-box transcription factors; PD-1, programmed cell death receptor ligand-1, IFN-γ, interferon γ. Continuous variables are expressed as the median and interquartile range or mean ± standard deviations (SD). *P* value for the comparison between Non-sepsis, Mild Sepsis and severe Sepsis.
