# Supplementary material for: Early Expression of Functional Markers on CD4+ T Cells Predicts Outcomes in ICU Patients With Sepsis
Source: Front Immunol. 2022 Jul 11;13:938538. doi: 10.3389/fimmu.2022.938538 (PMC9309518; doi:10.3389/fimmu.2022.938538)
Supplement: Supplementary file 1 [file DataSheet_1.zip › supplement table 2.docx]

**Supplement table 2, Characteristics of septic patients based upon 28-day mortality**

| **Variables** | **Overall**  **（n＝81）** | **Survivors**  **（n＝68）** | **Non-survivors**  **（n＝13）** | **P value** |
| --- | --- | --- | --- | --- |
| **Baseline characteristics** | | | | |
| Age (years) | 66.5 (22.8) | 65.5 (22.8) | 76 (9.8) | 0.055 |
| Sex (male: female) | 50: 31 | 42：26 | 8: 5 | 1.0 |
| APACHE II score | 16.52 ± 5.59 | 16.27 ± 5.33 | 19.25 ± 4.22 | 0.061 |
| **Comorbidities (n, %)** | | | | |
| Heart failure | 19（23.5） | 15（22.1） | 4（30.8） | 0.491 |
| COPD | 4（4.9） | 4（5.9） | 0（0） | 1.0 |
| Diabetic mellitus | 25（30.9） | 21（30.9） | 4（30.8） | 1.0 |
| Liver Cirrhosis | 3（3.7） | 3 (4.4) | 0 (0) | 1.0 |
| Tumor | 21（25.9） | 18（26.5） | 3（23.1） | 1.0 |
| Chronic renal failure | 19（23.5） | 15（22.1） | 4（30.8） | 0.491 |
| **Sites of infection (n, %)** | | | | |
| Lungs | 60（74.1） | 50（73.5） | 10（76.9） | 1.0 |
| BSI | 13（16.0） | 12（17.6） | 1(7.7) | 0.681 |
| Intra-abdominal | 20（24.7） | 14（20.6） | 6（46.2） | 0.076 |
| soft tissue | 6（7.4） | 5（7.4） | 1（7.7） | 1.0 |
| Others | 10（12.3） | 6（8.8） | 4（30.8） | 0.05 |
| **Pathogens (n, %)** | | | | |
| Bacteria | 67（82.7） | 56（82.4） | 11（84.6） | 1.0 |
| Fungal | 24（29.6） | 18（26.5） | 6（46.2） | 0.19 |
| Virus | 5（6.2） | 5（7.4） | 0（0） | 0.587 |
| Else | 4（4.9） | 4（5.9） | 0（0） | 1.0 |
| **Laboratory test at admission** | | | | |
| Creatinine (μmol/L) | 125.5 (93) | 128.5 (116) | 129.5 (108) | 0.949 |
| Albumin (g/L) | 32.06 ± 3.31 | 31.27 ± 3.29 | 31.25 ± 3.88 | 0.773 |
| TBiL (μmol/L) | 21 (30.7) | 19.7 (35.6) | 25.8 (27.9) | 0.163 |
| **Life-sustaining treatments (n, %)** | | | | |
| Mechanical ventilation | 72（88.9） | 61（89.7） | 11（84.6） | 0.632 |
| Need for vasopressor | 46（56.8） | 39（57.4） | 7（53.8） | 1.0 |
| Need for RRT | 24（29.6） | 19（27.9） | 5（38.5） | 0.513 |
| **Prognosis parameters** | | | | |
| ICU durations **(day)** | 10.5 (8.0) | 11 (11) | 12 (17.8) | 0.439 |
| ICU mortality **(n, %)** | 13 (16.0) | 2 (2.9) | 11 (84.6) | < 0.001 |
| Hospital durations **(day)** | 13 (18) | 15 (19.3) | 22.5 (18.3) | 0.847 |
| Hospital mortality **(n, %)** | 16 (19.8) | 4 (5.9) | 12 (92.3) | < 0.001 |

APACHE II, Acute Physiology and Chronic Health Evaluation II; COPD, chronic obstructive pulmonary disease; BSI, blood steam infection; TBIL, total bilirubin; RRT, renal replacement therapy; continuous variables are expressed as the median and interquartile range, other data are raw numbers (%). *P* value for the comparison between Non-sepsis, Mild Sepsis and severe Sepsis.
