# Supplementary material for: Early Expression of Functional Markers on CD4+ T Cells Predicts Outcomes in ICU Patients With Sepsis
Source: Front Immunol. 2022 Jul 11;13:938538. doi: 10.3389/fimmu.2022.938538 (PMC9309518; doi:10.3389/fimmu.2022.938538)
Supplement: Supplementary file 1 [file DataSheet_1.zip › supplement table 3.docx]

**Supplement table 3. ROC curve analysis of parameters in predicting 28-day mortality**

| **Parameter** | **Cut-off value** | **AUC** | **specificity** | **sensitivity** | **95% CI** | **P value** |
| --- | --- | --- | --- | --- | --- | --- |
| mTOR+/CD4+T% | 30.57 | 0.695 | 73.53 | 69.23 | 0.582 - 0.792 | 0.008 |
| PD1+CD4+T% | 22.46 | 0.71 | 85.29 | 53.85 | 0.599 - 0.806 | 0.01 |
| IFN-γ+/CD4+T% | 12.81 | 0.790 | 55.88 | 92.31 | 0.685 - 0.872 | < 0.001 |
| Apache II | 16 | 0.66 | 50 | 76.92 | 0.546 - 0.761 | 0.033 |

ROC, receiver operator characteristic; AUC, area under the curve; CI, confidence interval.
