# Supplementary material for: Early Expression of Functional Markers on CD4+ T Cells Predicts Outcomes in ICU Patients With Sepsis
Source: Front Immunol. 2022 Jul 11;13:938538. doi: 10.3389/fimmu.2022.938538 (PMC9309518; doi:10.3389/fimmu.2022.938538)
Supplement: Supplementary file 1 [file DataSheet_1.zip › supplement table 4.docx]

**Supplement table 4. The list of antibodies**

| **Antibody type** | **dose** | **clone** |
| --- | --- | --- |
| Brilliant Violet 510 labeled anti-CD3 | 5 μl | clone OKT3 |
| Brilliant Violet 570-labeled anti-CD4 | 5 μl | clone OKT4 |
| APC/Fire 750-labeled anti-CD8 | 5 μl | clone HIT8a |
| PerCP-eFluor 710-labeled anti-mTOR | 5 μl | clone MRRBY |
| PE-labeled anti-INF-γ | 5 μl | clone B27 |
| eFluor 450-labeled anti-Granzyme B | 5 μl | clone N4TL33 |
| PE/Dazzle 594-labeled anti-T-bet | 5 μl | clone 4B10 |
