# Supplementary figures and images for: Early Expression of Functional Markers on CD4+ T Cells Predicts Outcomes in ICU Patients With Sepsis
Source: Front Immunol. 2022 Jul 11;13:938538. doi: 10.3389/fimmu.2022.938538 (PMC9309518; doi:10.3389/fimmu.2022.938538)

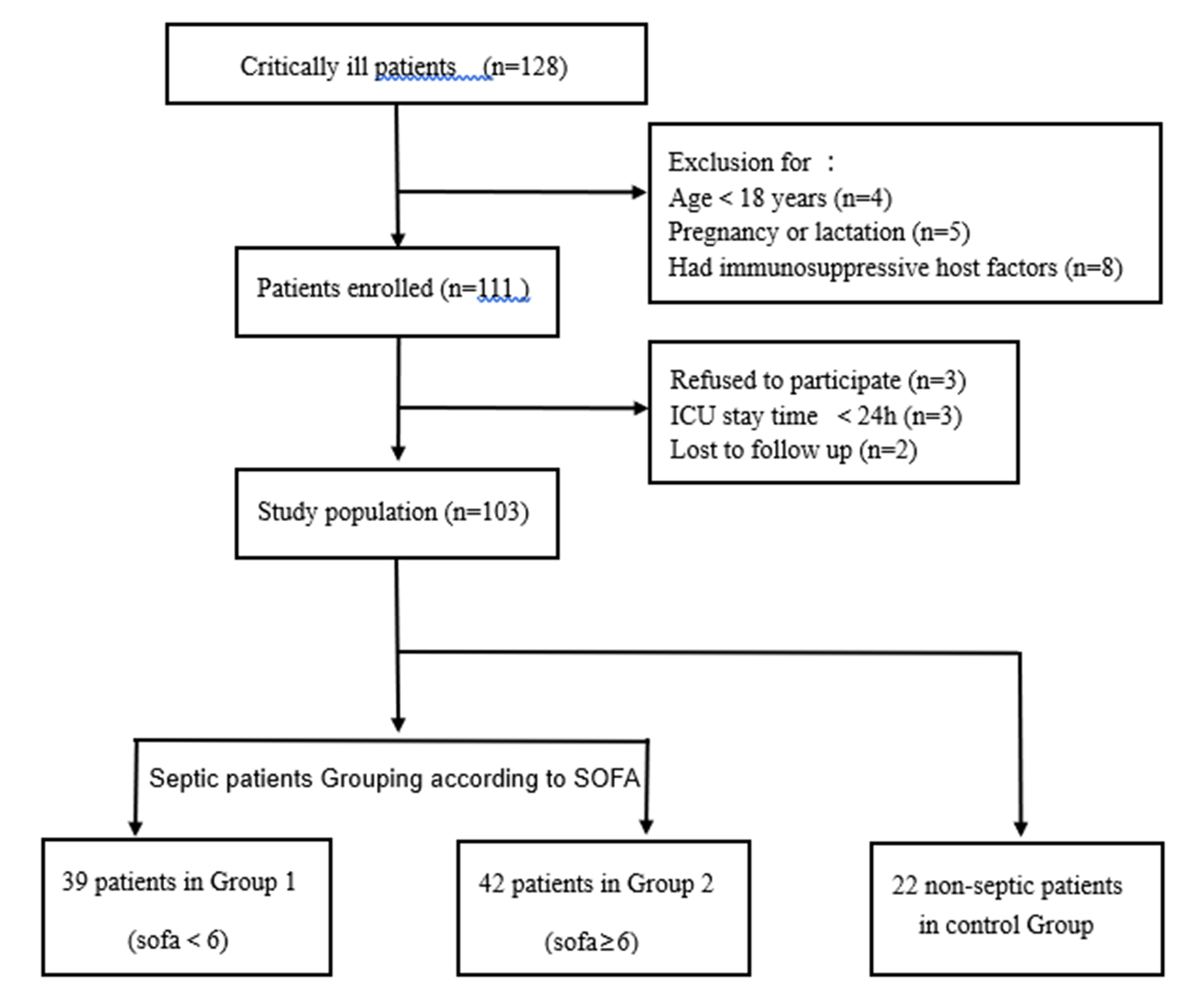

Supplement: Supplementary file 1 [file DataSheet_1.zip › Supplement figue1.png]

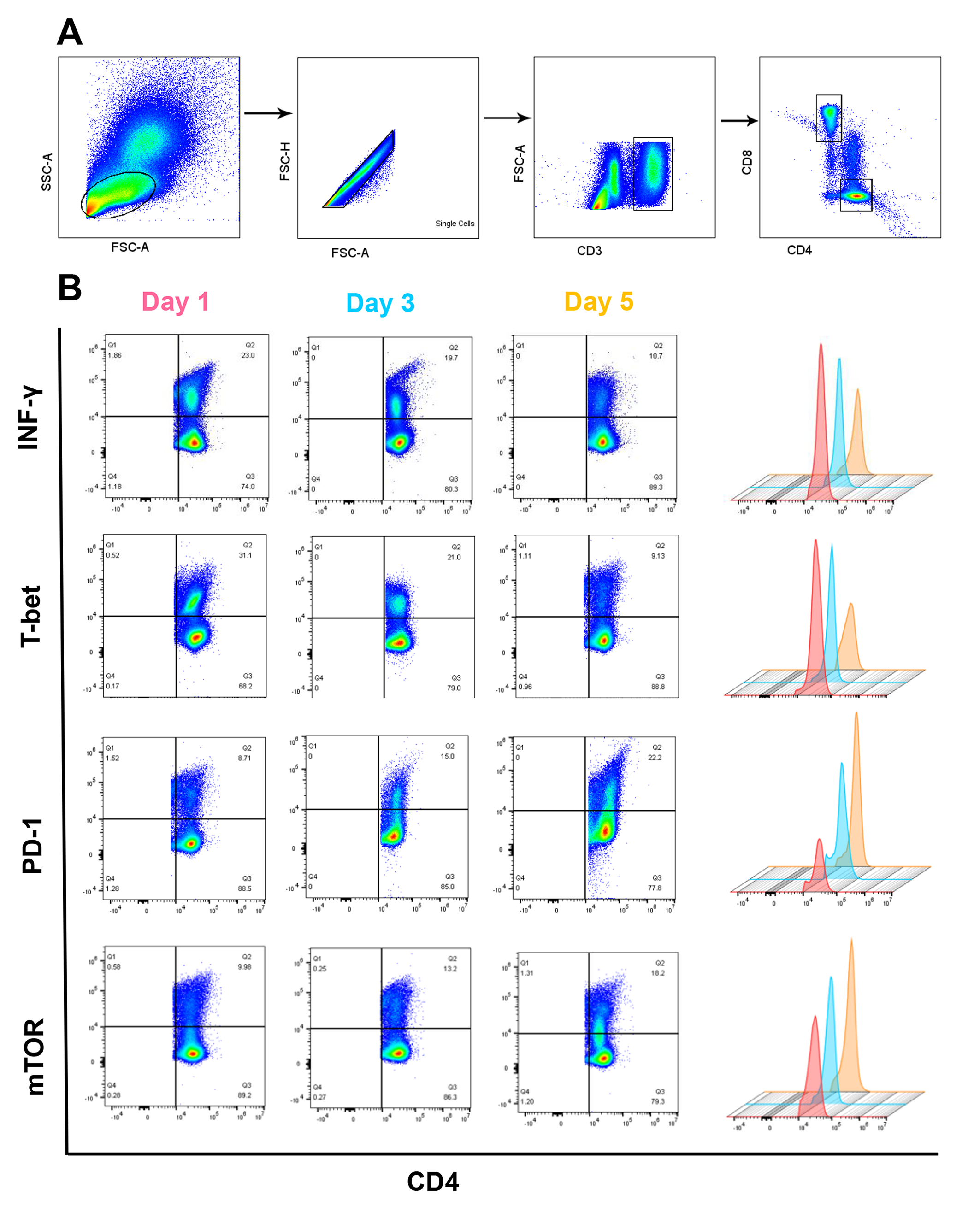

Supplement: Supplementary file 1 [file DataSheet_1.zip › Supplement figure 2..tif]

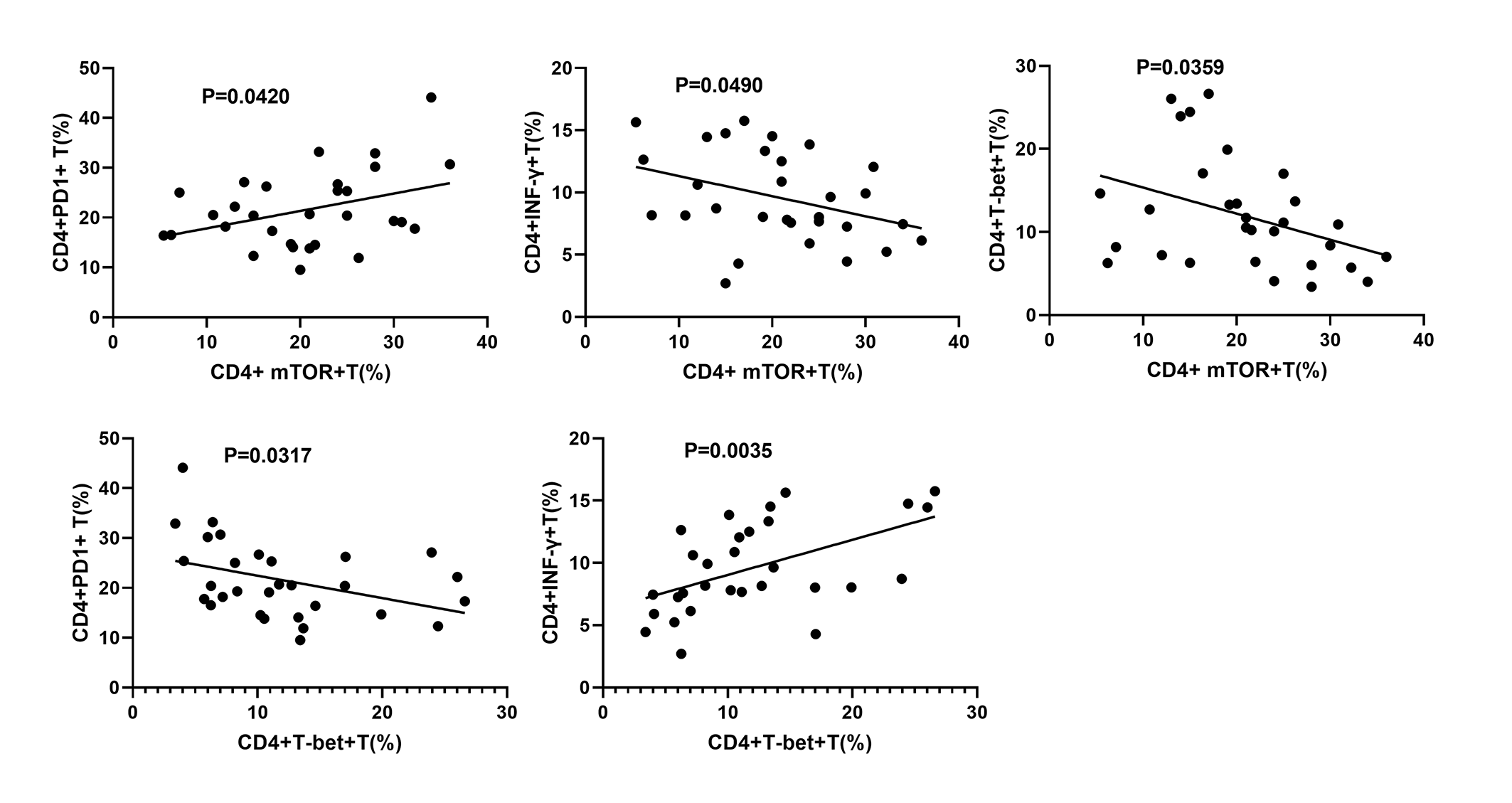

Supplement: Supplementary file 1 [file DataSheet_1.zip › Supplement figure 3..tif]
